# The Prevalence of Clinically Significant Ischemia in Patients Undergoing Percutaneous Coronary Intervention: A Report from the Multicenter Registry

**DOI:** 10.1371/journal.pone.0133568

**Published:** 2015-07-31

**Authors:** Jun Fujita, Shun Kohsaka, Ikuko Ueda, Taku Inohara, Yuichiro Maekawa, Akio Kawamura, Hideaki Kanazawa, Kentaro Hayashida, Ryota Tabei, Shugo Tohyama, Tomohisa Seki, Masahiro Suzuki, Motoaki Sano, Keiichi Fukuda

**Affiliations:** 1 Department of Cardiology, Keio University School of Medicine, 35 Shinanomachi, Shinjuku-ku, Tokyo, 160–8582, Japan; 2 National Hospital Organization Saitama National Hospital, 2–1 Suwa, Wako-shi, Saitama, 351–0102, Japan; Azienda Ospedaliero-Universitaria Careggi, ITALY

## Abstract

**Background:**

Myocardial perfusion scintigraphy (MPS) plays an important role in the evaluation and quantification of myocardial ischemia, and those with significant ischemia (SI) benefit most from revascularization procedures. This study aimed to identify the clinical factors and anatomical features associated with SI in patients with stable ischemic heart disease (SIHD).

**Methods and Results:**

Data were analyzed from 4197 SIHD patients undergoing percutaneous coronary intervention (PCI). Ischemia was based on MPS findings prior to PCI, with SI defined as an ischemic region of more than 10% of the total left ventricular area. Logistic regression analysis was performed to identify any clinical factors associated with SI. MPS was used to evaluate 1070 (25.5%) patients pre-procedurally. Patients with a history of heart failure, stroke, or anginal symptoms with Canadian Cardiovascular Society class 2 or more were more likely to have SI (odds ratio [OR] 1.63, p = 0.025, OR: 1.85, p = 0.009, and OR: 1.49, p = 0.003, respectively). When angiographic variables were considered, a proximal left anterior descending artery (pLAD) lesion was the sole factor associated with SI (OR: 1.45, p = 0.012). Importantly, those with SI had more in-hospital complications (p = 0.006), most notably post-PCI infarcts (p = 0.008).

**Conclusions:**

Patients’ background data, such as stronger anginal symptoms or a pLAD lesion, were associated with SI. Patients with SI must be treated with PCI to improve their long-term prognosis; however, procedure-related complications happen more frequently in SI patients than in non-SI patients. Physicians must give their full attention when performing the PCI procedure in SI patients to minimize their complication rate.

## Introduction

Percutaneous coronary intervention (PCI) is one of the most advanced therapies for coronary artery disease [1] and early revascularization has proven beneficial for patients with acute coronary syndromes [2]. However, the benefit of revascularization for stable ischemic heart disease (SIHD) remains controversial. Quantification of stress testing has been used to risk-stratify patients with SIHD [3], and the application of myocardial perfusion scintigraphy (MPS) with single-photon emission computed tomography (SPECT) in patients with SIHD has been studied intensively [4]. MPS is known to have both high sensitivity and specificity (90% and 80%, respectively) for ischemia and is useful for determination of the ischemic burden [5, 6]. Further, patients with an ischemic region comprising more than 10% of the left ventricular (LV) myocardium seem to benefit more from revascularization rather than conservative management [7, 8], and recent studies have advocated the importance of ischemia-guided PCI [9].

On the basis of these studies, the current evidence and guidelines suggest that the benefit of revascularization in SIHD is mostly limited to patients with moderate to severe ischemia (significant ischemia: SI) [10]. Therefore, it is pivotal to know the clinical features of patients with SI in order to manage their treatment plan. This study aimed to identify the clinical factors and anatomical features associated with SI in patients with SIHD who underwent PCI, and to compare their in-hospital outcomes to those of patients with no or mild ischemia (non-significant ischemia: non-SI).

## Methods

### Study population and design

The Japan Cardiovascular Database Keio interhospital Cardiovascular Studies (JCD-KiCS) is a large, ongoing, prospective, multicenter, cohort study designed to collect clinical background and outcome data on PCI patients. Data pertaining to approximately 150 variables are being collected. In this registry, participating hospitals have been instructed to record data from hospital visits for consecutive PCI patients and to register these data in an internet-based database. The database system performs checks to ensure that the reported data are complete and internally consistent. PCI performed using any commercially available coronary device may be included. A significant stenosis was defined more than 50% stenosis in left main trunk coronary artery, and 75% in the other vessels. The decision to perform PCI is based on the attending physicians’ clinical assessment of the patient. The study does not mandate specific interventional or surgical techniques, such as vascular access, or the use of a specific stent or closure device. The majority of the clinical variables in the JCD-KiCS were defined according to the National Cardiovascular Data Registry (NCDR), which is sponsored by the American College of Cardiology to conduct comparative research and determine the factors that lead to disparities in PCI management [11, 12].

### Information disclosure

Before the launch of the JCD-KiCS, information on the objectives of the study, its social significance, and an abstract were provided to register this clinical trial with the University Hospital Medical Information Network. This Network is recognized by the International Committee of Medical Journal Editors as an “acceptable registry,” according to a statement issued in September 2004 (UMIN R000005598).

### Study participants

Major teaching hospitals within the metropolitan Tokyo area were selected for the pilot phase of this study. The study protocol was approved by the Institutional Review Board, Keio University School of Medicine, and all patients provided written informed consent to their enrolment in our registry. In this registry, data have been collected since September 2008 from the 16 Japanese hospitals participating in the JCD-KiCS [13, 14]. All patients aged >18 years undergoing PCI in these hospitals were enrolled.

### Procedures and data collection

**Fig 1:**
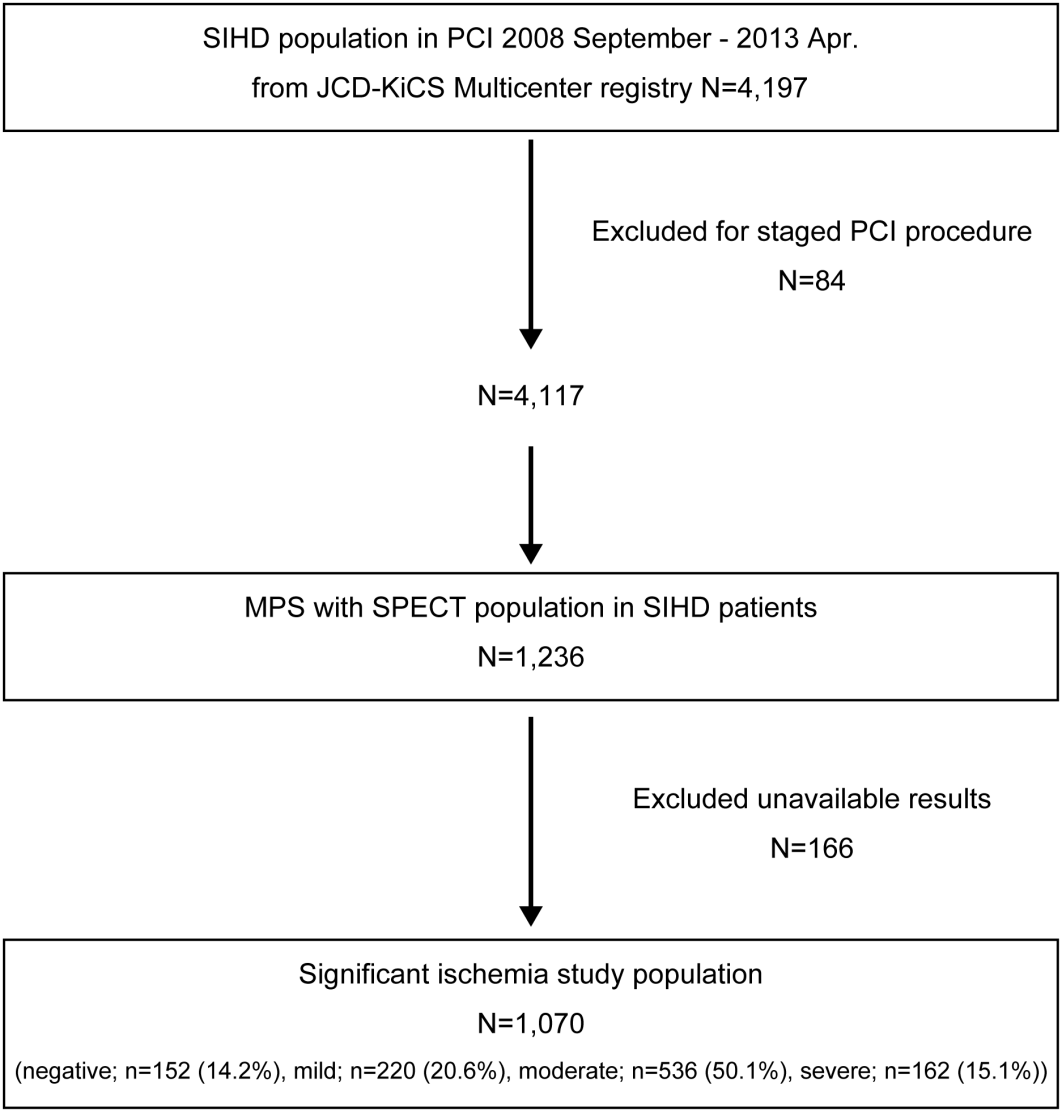
Flowchart of study progress. For the present study, out of a total of 4197 patients with stable ischemic heart disease (SIHD), 1236 underwent MPS. One hundred sixty-six patients were excluded because of unavailable data. The remained 1070 cases were analyzed. PCI = percutaneous coronary intervention; JCD-KiCS = Japan Cardiovascular Database-Keio Inter-hospital Cardiovascular Studies; MPS = myocardial perfusion scintigraphy; SPECT = single photon emission computed tomography; significant ischemia = moderate to severe ischemia.

Data were analyzed from the 4197 patients undergoing PCI for SIHD at one of the 16 Japanese hospitals participating in the JCD-KiCS between September 2008 and April 2013. SIHD was defined according to the patient’s presentation: patients who presented with no symptoms, or typical or atypical angina were included, whereas patients who presented with clinical symptoms or signs of acute coronary syndrome were excluded [10]. In addition, patients who had staged PCI during the same admission were excluded (n = 84). Of the 1236 patients who underwent MPS before PCI, 166 patients were excluded because their results were unavailable. Finally, a total of 1070 patients (25.5%) were evaluated by MPS (Fig 1). Before the PCI, the patients were categorized into two groups based on their MPS results: SI (at least moderate ischemia) and non-SI. A board-certified cardiologist visually interpreted the study as normal or abnormal, based on a review of all three standard cardiac projections, as well as the gated SPECT and raw image data. The SPECT studies were interpreted prospectively at the time of their completion, but later reviewed by a study investigator (also a board-certified nuclear cardiologist; SK) when the ischemic region was not quantified on the report. Quantification of total LV perfusion defect size, the extent of scar and ischemia, and LV ejection fraction was routinely performed. Based on previous prognostic studies, a severe and moderate ischemic LV perfusion defect size was prospectively defined as >15% and >10%, respectively [15]. The failure to document information was detected by the clinical coordinator and its input was mandated by the site data manager. Some patients in the SI study population had coronary computed tomography angiography (CTA) before or after MPS.

As a representative of anatomy-oriented PCIs, CTA-oriented patients were also evaluated for comparison. Of the 1,433 patients who underwent CTA before PCI, 707 who underwent concomitant ischemic evaluation and 32 with unavailable results were excluded. Overall, 694 patients (16.5%) were evaluated as the CTA study group (S1 Fig).

The study endpoints included in-hospital mortality, HF, cardiogenic shock, and other complications. Complications were defined as all complications, including severe dissection or coronary perforation, myocardial infarction (MI) after PCI, contrast-induced nephritis, cardiogenic shock or HF, cerebral bleeding or stroke, and bleeding complications. Heart failure (HF) was defined as physician documented or reported clinical symptoms of HF, such as unusual dyspnea on light exertion, recurrent dyspnea occurring in the supine position, fluid retention; or the description of rales, jugular venous distension, or pulmonary edema on physical examination; or pulmonary edema evident in chest radiographs and presumed to be associated with cardiac dysfunction. A low ejection fraction, without clinical evidence of HF, did not qualify as HF. Cardiogenic shock was defined as a sustained (>30 minutes) episode of systolic blood pressure <90 mm Hg, and/or a cardiac index of <2.2 L/min/m^2^ determined to be secondary to cardiac dysfunction, and/or the requirement for parenteral inotropic or vasopressor agents or mechanical support (e.g., intraaortic balloon pump, extracorporeal circulation, and ventricular assist devices) to maintain blood pressure and a cardiac index above the levels specified. Bleeding complications in this registry were defined as those requiring transfusion, prolonging hospital stay, and/or causing a decrease in hemoglobin of >3.0 g/dL [16]. Bleeding complications were further subdivided into puncture-site bleeding, retroperitoneal bleeding, gastrointestinal bleeding, genitourinary bleeding, or other bleeding.

### Data analyses

Continuous variables are expressed as means and standard deviations; categorical variables are expressed as percentages. Continuous variables were compared using Student’s t-test and differences between categorical variables were examined using a chi-squared test. A multiple logistic regression analysis was performed to determine the independent predictors of SI. Initially, the outcome variable was regressed on indicator variables that were available at the time of the admission (e.g., patient background); they were then regressed with additional variables that were available after the coronary angiogram (e.g., location of the lesion). All statistical calculations and analyses were performed using SPSS version 22 (SPSS, Chicago, IL, USA). P-values of <0.05 were considered statistically significant.

## Results

### Patient characteristics

The clinical characteristics and risk factors stratified by categories of MPS severity on SPECT are given in Table 1. Of the total of 1070 patients, 698 (65.2%) were determined by MPS to have SI, while 372 (34.8%) were non-SI. Overall, the mean age was 69 years, and 80% were males. The proportion with a body mass index (BMI) of >25 kg/m^2^ was approximately 40% in both groups.

**Table 1:** Patients’ characteristics.

	non-SI (N = 372)	SI (N = 698)	p Value
Age (years)	68.9 ± 9.2	69.0 ± 8.8	0.853
Male	295 (79.5)	564 (80.8)	0.614
Body mass index > 25kg/m2	143 (38.4)	286 (41.1)	0.4
Hypertension	303 (81.5)	577 (82.7)	0.621
Diabetes on insulin	43 (11.6)	96 (13.8)	0.305
Dyslipidemia	274 (73.7)	534 (76.6)	0.284
Current smoker	112 (30.1)	199 (28.6)	0.594
Myocardial infarction	125 (33.6)	242 (34.7)	0.726
Heart failure	32 (8.6)	101 (14.5)	0.005
Hemodialysis	21 (5.6)	47 (6.7)	0.487
Stroke	26 (7)	89 (12.8)	0.004
Peripheral artery disease	50 (13.4)	97 (13.9)	0.837
COPD	11 (3)	19 (2.7)	0.825
previous PCI	169 (45.4)	303 (43.4)	0.526
previous CABG	21 (5.6)	50 (7.2)	0.342
Family history of coronary artery disease	44 (12.4)	108 (15.9)	0.132
Angina symptom (CCS>2)	129 (35.3)	300 (44.1)	0.006
Heart failure symptom (>NYHAII)	21 (5.7)	43 (6.2)	0.734
CTA performance	143 (38.5)	192 (27.5)	<0.001

Values are mean ± standard deviation or n (%). non-SI, non-significant ischemia; SI, significant ischemia; COPD, chronic obstructive pulmonary disease; PCI, percutaneous coronary intervention; CABG, coronary artery bypass grafting; CCS, Canadian Cardiovascular Society classification of angina pectoris; NYHA, New York Heart Association functional classification; CTA, coronary computed tomography angiography.

Patients who had SI were significantly more likely than non-SI patients to have a history of HF (14.5% vs. 8.6%, p = 0.005), a history of stroke (12.8% vs. 7%, p = 0.004), anginal symptoms (CCS > class 2; 44.1% vs. 35.3%, p = 0.006), and were less likely to have undergone CTA (27.5% vs. 38.5%, p < 0.001).

The anatomical features of the patients’ coronary arteries were also stratified by categories of severity of MPS on SPECT, as shown in Table 2. A lesion in the proximal left anterior descending artery (pLAD) was seen more frequently in the patients with SI than in the non-SI patients (37.3 vs. 27.5%, p = 0.001). Patients with multiple vessel disease tended to have SI compared to those without (71.9% vs. 66.3%, P = 0.057).

**Table 2:** Angiographic characteristics

	non-SI (N = 372)	SI (N = 698)	p Value
LMT	29 (7.8)	63 (9.1)	0.489
proximal LAD	102 (27.5)	259 (37.3)	0.001
distal LAD	233 (62.8)	451 (64.9)	0.498
LCX	204 (55.1)	418 (60.1)	0.114
RCA	211 (57.3)	414 (59.5)	0.499
Multiple vessels	244 (66.3)	497 (71.9)	0.057

Values are n (%). non-SI, non-significant ischemia; SI, significant ischemia; LMT, left main trunk coronary artery; LAD, left anterior descending artery; LCX, left circumflex artery; RCA, right coronary artery.

### Clinical and angiographic predictors of SI

Based on the patients’ characteristics, statistically significant variables (a history of HF, a history of stroke, and CCS classes) were selected for the multivariate logistic regression model, together with age and sex. (Table 3). The presence of anginal symptoms of CCS class 2 or more (OR: 1.49, 95% CI: 1.14–1.95, p = 0.003), as well as a history of HF or stroke, were significantly associated with SI (OR: 1.63, 95% CI: 1.06–2.51, p = 0.025; OR: 1.85, 95% CI: 1.16–2.94, p = 0.009; respectively).

**Table 3:** Predictive factors for significant ischemia

Variables	Odds Ratio	95% Confidence Limits		p Value
Age	0.99	0.984	1.012	0.784
Male	1.24	0.895	1.729	0.194
History of HF	1.63	1.064	2.514	0.025
History of stroke	1.85	1.169	2.947	0.009
Angina symptom (CCS>2)	1.49	1.144	1.955	0.003

HF, heart failure; CCS, Canadian Cardiovascular Society Classification of angina pectoris.

The presence of pLAD occlusion was also strongly associated with SI (OR: 1.45, 95% CI: 1.08–1.95, p = 0.012) (Table 4). A history of HF or stroke, and the presence of anginal symptoms of the CCS class 2 or more were significantly correlated with SI (OR: 1.67, 95% CI: 1.08–2.58, p = 0.021; OR: 1.77, 95% CI: 1.11–2.82, p = 0.016; and OR: 1.44, 95% CI: 1.09–1.88, p = 0.008; respectively).

**Table 4:** Independent correlates of significant ischemia.

Variables	Odds Ratio	95% Confidence Limits		p Value
Age	0.997	0.983	1.012	0.702
Male	1.195	0.856	1.666	0.295
History of HF	1.67	1.08	2.583	0.021
History of stroke	1.774	1.114	2.823	0.016
Angina symptom (CCS>2)	1.44	1.099	1.888	0.008
proximal LAD	1.458	1.087	1.955	0.012
Multiple vessels	1.129	0.845	1.509	0.411

HF, heart failure; CCS, Canadian Cardiovascular Society Classification of angina pectoris; LAD, left anterior descending artery.

### Major complications after PCI

**Fig 2:**
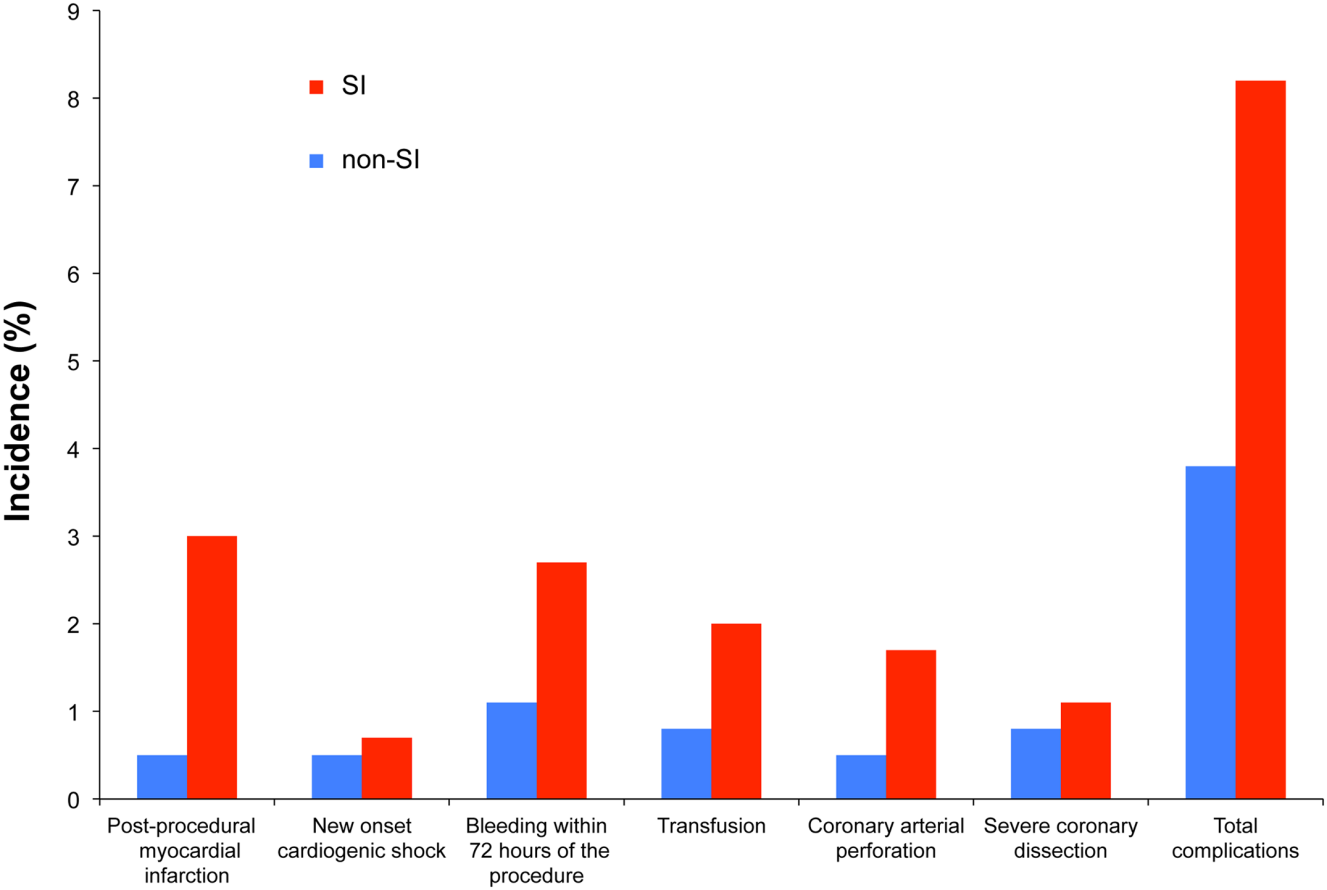
Correlation between significant ischemia and in-hospital complications. The incidence of major complications after percutaneous coronary intervention in patients with significant ischemia (SI) (red) and in those without SI (non-SI; negative or mild ischemia) (blue).

**Fig 3:**
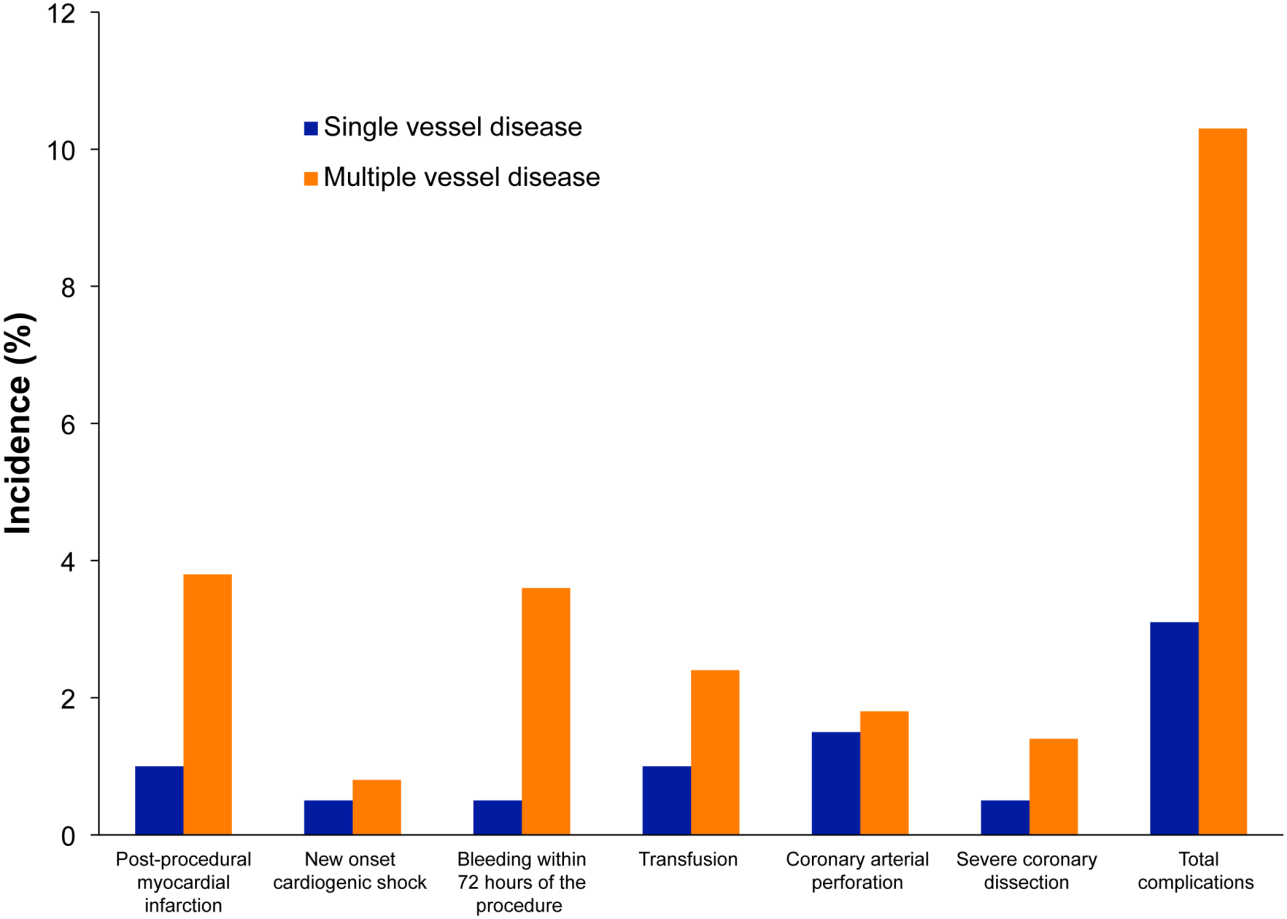
Correlation between multiple vessel disease in patients with significant ischemia (SI) and in-hospital complications. The incidence of major complications after percutaneous coronary intervention in patients with SI and single vessel disease (dark blue) or multiple vessel disease (orange).

In the evaluation of in-hospital outcomes, complications after PCI were also stratified by categories of severity of MPS on SPECT. The results are shown in Fig 2. Patients with SI had significantly more complications than non-SI patients (8.2% vs. 3.8%, p = 0.006), the most notable being post-procedural MI (3.0% vs. 0.5%, p = 0.008). Incomplete revascularization did not differ between the SI and non-SI patients (3.1% vs. 2.2%, respectively; p = 0.423). Patients with multiple vessel disease in the SI population had significantly higher in-hospital complications (10.3% vs. 3.1%, p = 0.002), especially post-procedural MI and bleeding (3.8% vs. 1.0%, p = 0.055; 3.6% vs. 0.5%, p = 0.025, respectively) (Fig 3).

The complication ratio was also compared with CTA-oriented PCI patients. CTA-oriented PCI patients had less hypertension, diabetes on insulin, dyslipidemia, and hemodialysis. They were also less likely to have a history of MI, HF, and PCI compared to MPS-oriented PCI patients (p < 0.001; S1 Table). However, they experienced significantly more complications (6.6%, p = 0.022), particularly post-procedural MIs (2.4%, p = 0.032) (S2 Fig).

## Discussion

Through the analysis of a contemporary multicenter PCI registry, we were able to identify some clinical and angiographic markers of SI: anginal symptoms of more than CCS class 2 and pLAD occlusion were the variables best correlated with SI. These clinical variables could aid in identifying patients who are likely to gain a true prognostic benefit from PCI. In addition, these results suggests that the importance of history taking with regard to angina pectoris should be emphasized once again, so that SI patients are not overlooked. Since the risk of complications after PCI is much higher in SI than in non-SI patients, caution is needed when making joint decisions about revascularization with actual SIHD patients.

The proof and evaluation of ischemia are necessary prerequisites for PCI, based on multiple outcome studies. The BARI-2 and COURAGE studies confirmed that optimal medical therapy had as good a prognosis as revascularization in low-risk SIHD patients [8, 17]. The presence of anginal symptoms has been known to be a significant predictor of a positive result in SPECT and is associated with early revascularization [7, 18]. Our study adds the notion that the severity of anginal symptoms according to the CCS scale has a cumulative predictive value for SI. A history of stroke was also a significant predictor of SI, probably because stroke and coronary artery disease share common risk factors [19]; in fact, cardiovascular events were the most frequent cause of death 3 to 5 years after the onset of the first stroke [20]. This is of particular importance since Asian subjects have been demonstrated to have higher mortality from stroke than non-hispanic whites [21].

Among the angiographic features related to SI, a lesion in the pLAD was the most strongly associated finding, which is reasonable since the LAD supplies more blood to the LV myocardium (40–50% of the total LV myocardium) than the rest of the coronary artery branches; in a previous study, pLAD stenosis caused approximately twice as large a defect on MPS as the other coronary arteries [22]. In accordance with the greater perfusion area, a pLAD lesion has also been noted as an important prognostic indictor [23]. In one classic study, a better 6-month outcome was observed in pLAD patients who underwent percutaneous transluminal coronary angiography (PTCA) compared to medical therapy [24].

In the modern era, PCI tends to be applied to higher risk patients [25]. In general, patients with SI benefit more from revascularization, while according to a sub-analysis of various studies, the use of PCI appears to be associated with improved outcomes [26]. The results of the present study suggested that patients with SI should be informed of their risk of PCI-related complications at the time of consent.

Bleeding is the most notorious complication observed in PCI procedures [27, 28], but the incidence of bleeding complications was not significantly higher in the SI group in our study (bleeding within 72 hours of the procedure: 2.7% vs. 1.1%, p = 0.077; transfusion: 2.0% vs. 0.8%, p = 0.135; for SI vs. non-SI, respectively). In this study, the most common complication was post-procedural MI, as indicated by an increase in creatine kinase (CK). In the balloon angioplasty era, an increase in CK was most strongly associated with increased late cardiac mortality following elective coronary artery intervention [29]. The five times greater the increase in CK-myocardial band post PCI, the higher the patients’ in-hospital clinical outcomes in the non-balloon device era [30]. The mechanism of a post-PCI-procedural MI is mostly due to side branch occlusion (proximal type) and microvascular injury (distal type) [31]. In our data, patients with SI had more pLAD lesions, which must be related to side branch occlusion involving a major septal branch and diagonal branches, and distal embolization. Moreover, patients with SI had more strokes and tended to have multivessel disease. These results coincided with data showing that post-PCI-procedural MI was strongly associated with systemic atherosclerosis and multivessel disease [30, 31].

Measures of either anatomic or ischemic burden are routinely used clinically to assess for indications of revascularization procedures in Japan. MPS has been found to be the most reliable modality for defining ischemic burden [3, 32]. Recently, CTA has been emerging as a major pre-procedural imaging modality for SIHD; indeed, in the present registry, the number of CTA examinations has been increasing since 2009. In our analysis, despite a lower risk of coronary artery disease in CTA-oriented PCI patients, the complication rate was equivalent to that of SI patients. This indicates that anatomical assessment alone may not suffice to predict in-hospital complications; functional assessment by MPS may more precisely risk stratify patients who undergo PCI. More recently, the PROMISE study reported that CTA-oriented PCI did not change clinical outcomes compared with MPS, and it led to more catheterization procedures [33]. Further studies are needed to justify these findings in the real-world practice setting.

### Limitations

Our study has the inherent limitation of its retrospective design, although the data were collected prospectively. The operators were not blinded to the clinical and laboratory information, including the MPS results. Because of the limited availability of data fields, data on nuclear species, stress protocol, and the usage of β-blockers and Ca blockers before MPS were not included in our database. Given the methodological limitations inherent to retrospective registry analyses, our data cannot establish a definite etiological link between SI and the increased risk of complications. Furthermore, only 25.5% of SIHD patients underwent MPS before PCI. Our findings need to be confirmed in larger multicenter trials. Currently, there is no randomized trial to define the effect of ischemia severity on patients’ prognosis. The prospective association with prognosis will be investigated by the ongoing “ISCHEMIA” outcome study: http://clinicaltrials.gov/show/NCT01471522.

## Conclusions

SI as determined by MPS remains important preprocedural information when performing revascularization procedures, and several items among the patients’ background information (stroke, HF, anginal symptoms, and/or pLAD lesion) were significantly correlated with SI. Patients with SI must be treated with PCI to improve their long-term prognosis, however procedure-related complications happen more frequently in SI patients than in non-SI patients. Physicians must give their full attention when performing the PCI procedure in SI patients to minimize their complication rate.

## Supporting Information

S1 Fig Flowchart of the study progress (CTA vs. MPS). In the coronary computed tomography angiography (CTA) study, 1,433 of 4,197 patients with stable ischemic heart disease (SIHD) underwent CTA. Seven hundred seven patients were excluded because of ischemic evaluation, and 32 were excluded because of unavailable data. The remaining 694 cases were analyzed. PCI = percutaneous coronary intervention; JCD-KiCS = Japan Cardiovascular Database-Keio Inter-hospital Cardiovascular Studies; MPS = myocardial perfusion scintigraphy.
(TIF)

S2 Fig Correlation between anatomy-oriented PCI and in-hospital complications. The incidence of major complications after PCI in CTA-oriented PCI patients (yellow), patients with SI (red), and those without SI (nonSI; negative or mild ischemia) (blue). PCI = percutaneous coronary intervention; CTA = computed tomography angiography; SI = significant ischemia.
(TIF)

S1 Table Patients’ characteristics (CTA vs MPS). CTA-oriented PCI patients had less hypertension, diabetes on insulin, dyslipidemia, and hemodialysis. They were also less likely to have a history of myocardial infarction, heart failure, and PCI compared to MPS-oriented PCI patients (p < 0.001). Values are mean ± standard deviation or n (%). CTA, coronary computed tomography angiography; MPS, myocardial perfusion scintigraphy; COPD, chronic obstructive pulmonary disease; PCI, percutaneous coronary intervention; CABG, coronary artery bypass grafting; CCS, Canadian Cardiovascular Society classification of angina pectoris; NYHA, New York Heart Association functional classification.
(DOCX)
