# Supplementary figures and images for: The Prevalence of Clinically Significant Ischemia in Patients Undergoing Percutaneous Coronary Intervention: A Report from the Multicenter Registry
Source: PLoS One. 2015 Jul 31;10(7):e0133568. doi: 10.1371/journal.pone.0133568 (PMC4521937; doi:10.1371/journal.pone.0133568)

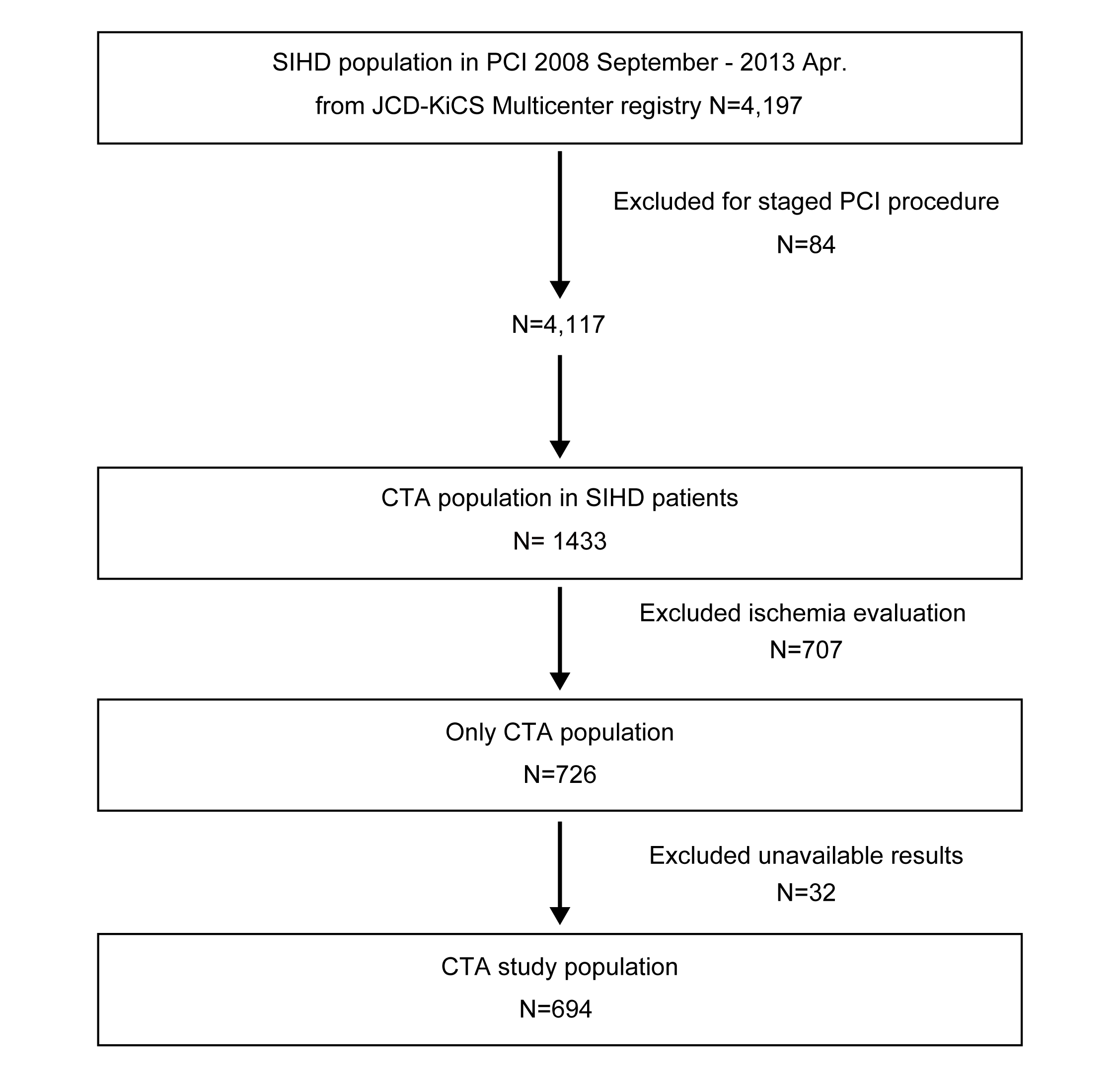

Supplement: S1 Fig — In the coronary computed tomography angiography (CTA) study, 1,433 of 4,197 patients with stable ischemic heart disease (SIHD) underwent CTA. Seven hundred seven patients were excluded because of ischemic evaluation, and 32 were excluded because of unavailable data. The remaining 694 cases were analyzed. PCI = percutaneous coronary intervention; JCD-KiCS = Japan Cardiovascular Database-Keio Inter-hospital Cardiovascular Studies; MPS = myocardial perfusion scintigraphy. (TIF) [file pone.0133568.s001.tif]

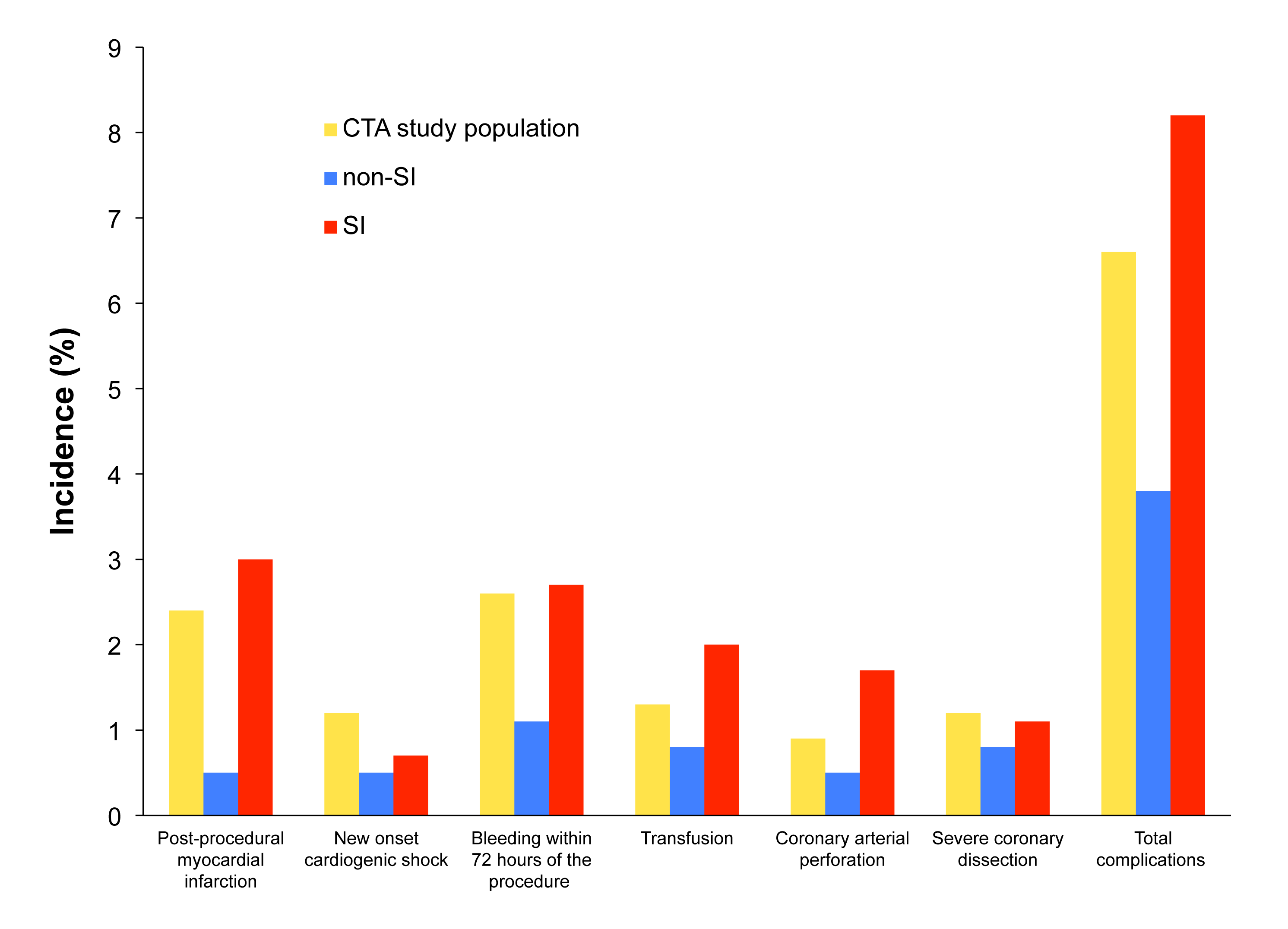

Supplement: S2 Fig — The incidence of major complications after PCI in CTA-oriented PCI patients (yellow), patients with SI (red), and those without SI (nonSI; negative or mild ischemia) (blue). PCI = percutaneous coronary intervention; CTA = computed tomography angiography; SI = significant ischemia. (TIF) [file pone.0133568.s002.tif]
