# Supplementary material for: The Prevalence of Clinically Significant Ischemia in Patients Undergoing Percutaneous Coronary Intervention: A Report from the Multicenter Registry
Source: PLoS One. 2015 Jul 31;10(7):e0133568. doi: 10.1371/journal.pone.0133568 (PMC4521937; doi:10.1371/journal.pone.0133568)
Supplement: S1 Table — CTA-oriented PCI patients had less hypertension, diabetes on insulin, dyslipidemia, and hemodialysis. They were also less likely to have a history of myocardial infarction, heart failure, and PCI compared to MPS-oriented PCI patients (p < 0.001). Values are mean ± standard deviation or n (%). CTA, coronary computed tomography angiography; MPS, myocardial perfusion scintigraphy; COPD, chronic obstructive pulmonary disease; PCI, percutaneous coronary intervention; CABG, coronary artery bypass grafting; CCS, Canadian Cardiovascular Society classification of angina pectoris; NYHA, New York Heart Association functional classification. (DOCX) [file pone.0133568.s003.docx]

Patients’ characteristics (CTA vs MPS)

|  | CTA study (N= 694) | MPS (N= 1,070) | p Value |
| --- | --- | --- | --- |
| Age (years) | 68.3 ± 10.2 | 69.1 ± 9.0 | 0.115 |
| Male | 568 (81.8) | 859 (80.4) | 0.437 |
| Body mass index > 25kg/m2 | 249 (36) | 429 (40.2) | 0.078 |
| Hypertension | 514 (74.2) | 880 (82.2) | <0.001 |
| Diabetes on insulin | 49 (7.1) | 139 (13) | <0.001 |
| Dyslipidemia | 434 (62.6) | 808 (75.6) | <0.001 |
| Current smoker | 210 (30.4) | 311 (29.1) | 0.56 |
| Myocardial infarction | 121 (17.4) | 367 (34.3) | <0.001 |
| Heart failure | 37 (5.3) | 133 (12.4) | <0.001 |
| Hemodialysis | 16 (2.3) | 68 (6.4) | <0.001 |
| Stroke | 80 (11.5) | 115 (10.7) | 0.603 |
| Peripheral artery disease | 75 (10.8) | 147 (13.7) | 0.07 |
| COPD | 24 (3.5) | 30 (2.8) | 0.436 |
| previous PCI | 170 (24.5) | 472 (44.1) | <0.001 |
| previous CABG | 45 (6.5) | 71 (6.6) | 0.906 |
| Family history of coronary artery disease | 54 (8.2) | 152 (14.7) | <0.001 |
| Angina symptom (CCS>2) | 333 (48) | 429 (41.1) | 0.004 |
| Heart failure symptom (>NYHAⅡ) | 19 (2.7) | 64 (6) | 0.002 |
| CTA performance | 694 (100) | 335 (31.4) | <0.001 |
| MPS performance | 0 (0) | 1070 (100) | <0.001 |

CTA-oriented PCI patients had less hypertension, diabetes on insulin, dyslipidemia, and hemodialysis. They were also less likely to have a history of myocardial infarction, heart failure, and PCI compared to MPS-oriented PCI patients (p < 0.001). Values are mean ± standard deviation or n (%). CTA, coronary computed tomography angiography; MPS, myocardial perfusion scintigraphy; COPD, chronic obstructive pulmonary disease; PCI, percutaneous coronary intervention; CABG, coronary artery bypass grafting; CCS, Canadian Cardiovascular Society classification of angina pectoris; NYHA, New York Heart Association functional classification.
